# Pioneer Transcription Factors: The First Domino in Zygotic Genome Activation

**DOI:** 10.3390/biom14060720

**Published:** 2024-06-18

**Authors:** Bo Fu, Hong Ma, Di Liu

**Affiliations:** 1 Institute of Animal Husbandry, HeiLongJiang Academy of Agricultural Sciences, Harbin 150086, China; fubo@haas.cn (B.F.); hongma@haas.cn (H.M.); 2 Key Laboratory of Combining Farming and Animal Husbandry, Ministry of Agriculture and Rural Affairs, Harbin 150086, China

**Keywords:** pioneer transcription factors, preimplantation embryo, zygotic genome activation, retrotransposons

## Abstract

Zygotic genome activation (ZGA) is a pivotal event in mammalian embryogenesis, marking the transition from maternal to zygotic control of development. During the ZGA process that is characterized by the intricate cascade of gene expression, who tipped the first domino in a meticulously arranged sequence is a subject of paramount interest. Recently, Dux, Obox and Nr5a2 were identified as pioneer transcription factors that reside at the top of transcriptional hierarchy. Through co-option of retrotransposon elements as hubs for transcriptional activation, these pioneer transcription factors rewire the gene regulatory network, thus initiating ZGA. In this review, we provide a snapshot of the mechanisms underlying the functions of these pioneer transcription factors. We propose that ZGA is the starting point where the embryo’s own genome begins to influence development trajectory, therefore in-depth dissecting the functions of pioneer transcription factors during ZGA will form a cornerstone of our understanding for early embryonic development, which will pave the way for advancing our grasp of mammalian developmental biology and optimizing in vitro production (IVP) techniques.

## 1. Introduction

Following fertilization, the genome within the totipotent embryo remains transcriptionally silent, then initiates bursts of transcription, known as zygotic genome activation (ZGA). ZGA signifies the developmental governance from maternal genetic materials to zygotic control. This transition encompasses not only the degradation of maternal constituents but also the activation of the zygotic genome across two or more distinct transcriptional phases. Taking the early mouse embryo as an example, ZGA begins with the minor ZGA, which occurs in the one-cell stage embryo, then followed by the major ZGA, which is more pronounced and occurs at the two-cell stage. In fact, the timing and progression of ZGA vary across species, which reflects the diversity in embryonic development strategies employed by different mammals [1,2]. The ZGA process initiates a series of tightly regulated molecular events that are essential for the progression of embryogenesis. In particular, chromatin remodeling occurs during ZGA, which facilitates the accessibility of the embryonic genome to the transcriptional machinery [3,4]. Within this complex transcriptional machinery, transcription factors, particularly a distinct subgroup known as pioneer transcriptional actors, play a pivotal role.

In the architectural configuration of the nucleosome, genomic DNA is wrapped around an octameric assembly of histones, presenting a formidable impediment to the binding of transcription factors. While architectural configuration of the nucleosome is crucial for the organization and integrity of the genome, this structural conformation poses a significant challenge to transcriptional activation processes, including those associated with ZGA [5]. Pioneer transcription factors, unlike other transcription factors that require accessible chromatin, can bind directly to nucleosomal DNA within compacted chromatin, thereby initiating chromatin remodeling and enabling gene expression. The term “pioneer” refers to transcription factors that meet specific criteria: they bind to their (partial) motifs within closed chromatin, are essential for local chromatin opening in vivo, and can bind to nucleosomes in vitro [6].

Pioneer transcription factors discussed in this review, including Dux, Obox and Nr5a2, are endowed with the unique capability to recognize and bind to specific sequences within their target motifs amidst the compact architecture of closed chromatin. This initial docking phenomenon is pivotal, as it triggers a sequence of chromatin remodeling activities that culminate in decondensation of chromatin. These pioneer transcription factors exhibit remarkable proficiency in infiltrating chromatin territories typically characterized by their repressive state within a living organism, catalyzing the transition of chromatin from an inaccessible to an open state within the physiological milieu in vivo. Such a transformative action on the chromatin landscape is mediated through diverse mechanisms, with each contributing to the facilitation of a more transcriptionally permissive environment [7,8,9,10,11]. Accordingly, it has been believed that this intrinsic attribute of these pioneer transcription factors to act as a trigger of chromatin accessibility is particularly critical in initiating the transcriptional awakening of the zygote. 

Currently, the elucidation of ZGA mechanisms within mammalian species remains comparatively nascent, especially when compared to the extensive knowledge amassed in model organisms such as fruit flies and zebrafish, whose pioneer transcription factors have already been subject to detailed study [12,13,14,15,16]; and the pioneer transcription factors for triggering ZGA in mammals still remain elusive. Fascinatingly, recent investigations have illuminated the complexity of transcriptional regulation in mammalian embryonic development, revealing that not only a single, but multiple pioneer transcription factors (including Dux, Obox and Nr5a2) bind to the genome to open chromatin, thereby triggering the initiation of ZGA [17,18,19,20,21]. Surprisingly, all these three pioneer transcription factors use retrotransposons as hubs to control ZGA gene expression, thus initiating the ZGA process. In this review, we summarized how Dux, Obox and Nr5a2 function as activators of ZGA in mammals and outlined the interaction between these pioneer transcription factors and retrotransposons. We anticipate that further elucidation of the mechanisms by which these pioneer transcription factors operate will offer a novel perspective for further understanding the ZGA process.

## 2. The Role of Dux in ZGA

Dux in mice or DUX4 in humans, the double homeobox (DUX) gene family members that are conserved in mammals, have been identified as a pioneer transcription factor that initiates ZGA. Using nanopore-based ultra-long read sequencing, Mitsuhashi et al. revealed that *DUX4* gene loci are located within the 3.3-kb repeats of the D4Z4 array at the subtelomeric region of chromosome 4q35 in human [22]. The results derived from DNA fluorescence in situ hybridization (FISH) also confirmed that there is a hybridization signal of *Dux* repeats on chromosome 10 in mice [23]. The double homeobox gene, such as DUX4 tandem repeats located within pericentromeric regions, generally carries characteristic heterochromatin, which serves to repress expression in the vast majority of somatic cell types. Once the pathological misexpression of *DUX4* from these tandem repeats occurs, the aberrant expression usually results in the transcriptional activation of the early embryonic program, and then causes facioscapulohumeral muscular dystrophy (FSHD) [24,25]. However, in the developmental context, mouse DUX and its human counterpart, DUX4, are both expressed before ZGA in their respective species [19,26]. Dux expression rapidly increases after fertilization and peaks at the two-cell stage. This temporal expression pattern indicates that the transient expression of Dux may underscore a pivotal role in the orchestration of ZGA [27]. For instance, with regard to somatic cell nuclear transfer (SCNT) embryos where the nucleus of a somatic cell was transferred into an enucleated oocyte to produce an embryo that is genetically identical to the donor cell, Dux is capable of rectifying aberrant H3K9ac acetylation and inducing local chromatin relaxation in SCNT embryos, thereby ensuring appropriate two-cell genome activation. Generally, abnormal H3K9ac patterns lead to diminished activation of the genome at the two-cell stage during the development of SCNT embryos. Nonetheless, overexpression of Dux corrected the aberrant H3K9ac signal at its target sites, thereby facilitating proper activation of the two-cell stage genome and improving SCNT efficiency. In addition, Yang et al. confirmed that the developmental advancements facilitated by TSA are notably hindered in the absence of Dux during the development of SCNT embryos, which also indicates that the rescue efficiency by TSA largely relies on Dux activation [28].

Investigations into the functional domains of Dux exhibit its structural complexity and reveal the critical domains responsible for its distinct regulatory actions, further substantiating the versatile roles of Dux in embryonic transcriptional regulation during ZGA. The mouse Dux protein, akin to the human DUX4 protein, comprises three functional domains. In detail, two adjacent homeodomains are located in the N-terminal (amino acids 29–72 and 113–166) and a singular domain is located in the C-terminal (amino acids 626–674), leaving amino acids 167–625 of Dux in the intermediate region. Huang et al. confirmed that Dux and DUX4 contain conserved N-terminal double homeodomains that are indispensable for its nuclear localization and any single homeodomain is also sufficient to show the nuclear location with the entire C-terminus. In addition, Huang et al. found that the intermediate region (AA167–625) of the Dux protein was responsible for activating the transcription of Zscan4 and MERVL [29]. The terminal 49 amino acids at the C-terminus of the mouse Dux protein exhibit remarkable similarity to the corresponding C-terminal domain of the human DUX4 protein. This structural conservation underscores a potential evolutionary parallel in their functional roles, indicating that these C-terminal sequences may harbor critical transcriptional regulation capabilities [23]. Recently, Christina et al. identified 5 approximately 100 amino acid repeats followed by a single 14 amino acid highly-acidic tail in the C-terminus of Dux. Mechanistically, there exists cooperativity between active repeats and the acidic tail, which may facilitate cofactor recruitment, Dux-mediated opening of targets and transcription [30]. Concerning the role of DUX4 in facilitating chromatin accessibility for transcription, DUX4 also orchestrates chromatin dynamics through its C-terminal domain, thereby influencing gene expression. In more detail, DUX4 recruits the histone acetyltransferases p300/CBP to its target genes. This recruitment leads to increased H3K27 acetylation at these sites, as indicated by chromatin immunoprecipitation experiments. Specifically, it was observed that DUX4 binding resulted in significant acetylation changes at the ZSCAN4 locus, with a marked increase in H3K27ac flanking the DUX4 binding site. This implies a reorganization of the nucleosome positioning around these loci. Furthermore, DUX4 is capable of binding to nucleosome-occupied DNA and facilitating chromatin remodeling. This is evidenced by the displacement of nucleosomes, particularly at previously inaccessible sites, following DUX4 binding. ChIP-seq data also reveals that DUX4 binding correlates with histone H3 depletion and increased H3K27 acetylation at target loci, suggesting a role in nucleosome repositioning [31]. Although DUX4 protein recruits the histone acetyltransferase P300 via its C-terminus and induces local chromatin opening, indicating that DUX4 may act as a pioneer factor, the direct binding of DUX4 to nucleosomes in vitro remains unconfirmed. Despite Dux’s paramount position within the transcriptional regulatory hierarchy, we should also bear in mind that ZGA may be redundantly driven by other pioneer transcription factors. In Dux-deficient mouse embryo models, a minority of Dux target genes were affected, and the knockout embryos did not arrest at the two-cell stage but survived to adulthood, albeit with diminished developmental capacities [32,33,34]. It is well known that genes exhibiting sequence homology may display genetic redundancy [35], and multiple functionally overlapping factors typically aim collectively toward a teleological objective in order to counteract sporadic mutations or defects [36,37]. Thus, it is reasonable to believe that such redundancy may represent an evolutionary adaptation or a fail-safe mechanism to guarantee the initiation of ZGA.

## 3. The Role of Obox in ZGA

Given that Dux is a homeobox gene, researchers are diligently investigating additional homeobox genes with the potential to trigger ZGA. Recently, Obox, a member of the homeobox gene family, has also been identified as a pioneer transcription factor that is functionally redundant to DUX during ZGA [20,38]. *Obox* genes, including Obox4, maps to proximal chromosome 7 in mouse [39]. Initially, both transcripts and translation levels of Obox4 are low within oocytes. During ZGA, Obox4 and its array of pseudogenes (Obox4-ps) exhibit notable dynamic in expression: the expression increased dramatically in early-two-cell embryos, then quickly declined in late-two-cell embryos [20]. The Obox4 protein is divided into three distinct regions: the N-terminal region, the central homeodomain (HD) region and the C-terminal region. Using a green fluorescent protein (GFP) fusion expression system, Park et al. revealed that intact HD region of Obox4 which harbors the presumptive DNA-binding motif essential for its transcription factor activity, may be responsible for the nuclear localization of Obox4 [40].

It is commonly acknowledged that ZGA in mice is marked by the pronounced upregulation of genes that are uniquely expressed during the two-cell (2C) stage, a significant number of which have co-opted MERVL long terminal repeats (LTRs) as promoters [41]. Thus, it is natural to raise the question whether Obox4 has the potential to trigger ZGA by activating MERVL, akin to the effects triggered by Dux. Through establishing a 2C::tdTomato reporter mESC line with the transgenic tdTomato red fluorescence protein driven by MERVL 5’-LTR, Guo et al. demonstrated that the ectopic expression of Obox4 led to a significant increase in tdTomato expression, mirroring the effect observed with Dux, thus highlighting the instrumental function Obox4 serves in the orchestration of ZGA [37]. Unexpectedly, when Obox4 genetic knockout mESCs were used as nuclear donor cells for SCNT, 54.4% of the reconstructed embryos still developed to the blastocyst stage. In addition, a single knockdown of Obox4 resulted in moderate developmental retardation in zygotes, with almost 50% of embryos advancing to the blastocyst stage. Nonetheless, the combined knockdown of Obox4 and Dux significantly hindered the formation of blastocysts, causing over 80% of the embryos to degenerate or stop developing before reaching the four-cell stage, and fewer than 20% displayed a morula-like structure. Presence of Obox4 can overcome the developmental arrest of Obox4/Dux double knockdown embryos. While RNA sequencing of two-cell stage embryos showed more severe dysregulation of two-cell specific genes and transposable elements due to the double knockdown of Obox4 and Dux compared to a single knockdown, reintroducing Obox4 can rectify the dysregulated transcriptome caused by the double knockdown. It should be noted that the knockout of the entire Obox cluster (including Obox1, Obox2, Obox3/4, Obox5, and Obox7) resulted in a 2–4 cell arrest in embryos. This phenomenon indicates that although Obox4 plays a redundant role in the absence of Dux to a certain extent, the functions of the Obox proteins cannot be fully compensated by Dux, as the complete knockout of the Obox cluster caused severe developmental arrest despite Dux’s presence [20,38].

In addition to Obox4, Obox3 also plays a critical role in activating ZGA. Obox3 interacts with specific repetitive elements such as MERVL long terminal repeats. Using ChIP-seq to detect Obox3 binding sites across the genome, Sakamoto et al. discovered that Obox3 primarily binds to promoters of ZGA genes, facilitating RNA Polymerase II pre-configuration at these sites. In addition, Obox3 significantly enhances the acquisition of totipotency in somatic cell nuclear transfer (SCNT) embryos. Sakamoto et al. demonstrated that injection of Obox3 mRNA in SCNT embryos significantly increases the success rate of SCNT embryos reaching the blastocyst stage. This process is accompanied by the restorative activation of ZGA genes and specific repetitive elements such as MERVL, indicating that Obox3 plays a crucial role in correcting abnormal transcription programs in SCNT embryos [42].

From a mechanistic perspective, Obox guides the timely pre-configuration of Pol II and chromatin accessibility at regulatory elements, which initiates the activation of Pol II targets and triggers ZGA. During mouse ZGA, Pol II undergoes “loading, pre-configuration, and synthesis” phases [43], and Pol II binding correlates with CG density. In more detail, although CG-rich promoters were inherently accessible for Pol II loading [44], the Pol II peaks specific to late-two-cell embryos, characterized by their low CG content, require the involvement of additional transcription factors [20]. In late-two-cell-specific Pol II targets, the Obox motif is the most enriched motif, and Obox protein exhibits the highest expression among the inferred transcription factors; furthermore, Ji et al., through performing Stacc-seq, confirmed that Obox proteins are responsible for recruiting RNA polymerase II to CG-poor promoters of ZGA genes in two-cell stage embryos [20]. Considering that the failure to open promoters and enhancers correlates with the downregulation of nearby ZGA genes, this phenomenon suggests that Obox proteins facilitate chromatin opening at regulatory elements and guide the pre-configuration of Pol II at these sites. Consequently, the absence of Obox leads to defective ZGA [20]. Intriguingly, based on ATAC-seq results, it was shown that active enhancers, defined by distal H3K27ac, exhibited substantial decreases in chromatin accessibility in *Obox* maternal-zygotic KO late-two-cell embryos, indicating that Obox depletion decreased chromatin accessibility at the late-two-cell-specific Pol II binding sites [20]. Although Obox may be involved in chromatin opening, whether Obox proteins possess all the characteristics of pioneer factors remains an open question. Collectively, Obox proteins are instrumental in enhancing chromatin accessibility at CG-poor promoters and enhancers and orchestrating the temporal pre-configuration of RNA polymerase II, thereby facilitating the initiation of transcriptional activation of RNA polymerase II-dependent genes, which pave the way for onset of ZGA.

## 4. The Role of Nr5a2 in ZGA

Nuclear receptor subfamily 5 group A member 2 (Nr5a2), also known as liver receptor homolog-1 (LRH-1), is a zinc-finger transcription factor that belongs to the orphan nuclear receptor superfamily. Mouse Nr5a2, the ortholog of NR5A2 in human, maps to chromosome 7 in mouse. Previous research has shown that Nr5a2 is expressed in liver, exocrine pancreas, intestinal crypts and ovaries in adult mammals [45,46,47,48]. Recently, experimental evidence derived from immunofluorescence has shown that although Nr5a2 exhibits very low expression in GV (germinal vesicle) oocytes, it is significantly upregulated in the two-cell stage embryos [21]. Thus, although it is known that Nr5a2 regulates cholesterol and bile acid homeostasis, as well as cholesterol delivery and steroid production [49,50,51,52], we raise the question of whether it also acts as a candidate ZGA regulator.

To ascertain the potential role of Nr5a2 in early embryonic development, particularly in the context of ZGA, the developmental potential of fertilized embryos was monitored. SR1848, a chemical inhibitor, causes Nr5a2 to move from the nucleus to the cytoplasm, thereby preventing it from activating the transcription of its target genes. To specifically explore the function of Nr5a2, SR1848 was used [53,54]. When embryos were exposed to SR1848 commencing at 6 h post fertilization (hpf), these embryos failed to develop into blastocysts and exhibited extensive fragmentation. These embryos starkly contrast to control embryos, corresponding to Nr5a2’s role in sustaining naïve pluripotency in mouse ES cells [55]. Intriguingly, transient inhibition of Nr5a2 from 6 to 36 hpf led to an arrest at the two-cell stage, while embryos treated with SR1848 beginning at 36 hpf (two-cell stage) manifested a less severe phenotype than those treated from 6 hpf. These phenomena indicated that Nr5a2’s activity is crucial between fertilization and the two-cell stage, coinciding with the occurrence of ZGA [21]. Subsequently, to explore whether Nr5a2 initiates the onset of ZGA, Gassler et al. utilized single-molecule FISH analysis algorithms to quantify the de novo expression of ZGA genes. They treated zygotes with SR1848, then observed a dose-dependent reduction in nascent ZGA transcripts [21]. Furthermore, using the CUT&Tag technique optimized for ultra-low input samples, Gassler et al. also assessed whether Nr5a2 actively binds in the vicinity of ZGA gene regions in two-cell embryos. In detail, using specific antibodies against Nr5a2, CUT&Tag results indicated a significant enrichment of Nr5a2 within these regions; and the motifs emerging from CUT&Tag peaks were characterized by an (A/G) (A/G) T sequence positioned upstream of the consensus sequences, which was also detected in a motif embedded in the *SINE B1/Alu 5YR* upstream of ZGA genes. This suggests that SINE B1 retrotransposons may serve as critical regulatory targets for Nr5a2 during the onset of ZGA [21]. Given the published ATAC-seq and ChIP-seq data (including histone modification H3K4me3 and H3K27ac) [56,57], it was also discovered that Nr5a2 binds to open chromatin, preferentially targeting enhancers in two-cell embryos [21].

Mechanistically, it is logical to pose the question of whether Nr5a2 performs its biological functions by promoting chromatin accessibility. Based on ChARM (Chromatin Accessibility Revealed by Microscopy) technology, both knockdown studies and chemical inhibition experiments confirmed that Nr5a2 is positively correlated with chromatin accessibility in early embryonic development; notably, treatment with SR1848 led to a substantial reduction in chromatin accessibility across a majority of Nr5a2 motifs. At the same time, Nr5a2 exhibits motif-dependent binding to nucleosomal DNA, preferentially at entry–exit sites, similar to known pioneer factors. Nr5a2’s interactions with nucleosomal DNA are both specific and competitive, which also align with the characteristics of pioneer transcription factors. Collectively, the combination of genome-wide binding profiles, chromatin accessibility assays and in vitro nucleosome binding has proven that Nr5a2 exhibits properties consistent with pioneer transcription factors [21]. Recently, Kobayashi et al. further demonstrated the mechanism underlying how Nr5a2 engages with nucleosomes to exert its pioneer activity [58]. The crystallographic analysis of the human NR5A2-DNA complex revealed that the α-helix of the zinc finger domain binds to the major groove, while the CTE (carboxy-terminal extension) binds to the minor groove [59]. Nr5a2, acting as a monomer, specifically binds to the DNA motif on both naked DNA and nucleosomes [21,60]. Through presenting a cryo-electron microscopy structure of human NR5A2 bound to a nucleosome, Kobayashi et al. observed the CTE loop of the NR5A2 DNA-binding domain interacts competitively with a DNA minor groove anchor of the nucleosome, facilitating the release of entry–exit site DNA, corresponding to the model in which histone–DNA interactions are compensated with pioneer transcription factor–DNA interactions [58,61]. Additionally, while the D159 residue of the CTE is not essential for DNA binding, it is crucial for maintaining stable nucleosome association and continuous DNA “unwrapping” [61]. Importantly, the RGGR motif within the CTE loop is a common characteristic among members of the NR5A and NR3B orphan nuclear receptor families, indicating that competition in the minor groove mediated by the CTE loop may be a conserved mechanism in these transcription factors [59].

Despite the conclusions from the study by Gassler et al. suggesting that Nr5a2 plays a certain role in the ZGA process, we shall also bear in mind that the role of Nr5a2 in ZGA remains a topic of ongoing debate. Recent studies utilizing more stringent genetic approaches have challenged these findings. For instance, Lai et al. demonstrated that Nr5a2 is largely dispensable for global ZGA but is essential for the 4–8 cell stage gene program.

Surprisingly, utilizing comprehensive ATAC-seq and CUT&RUN techniques to profile Nr5a2 binding, Lai et al. obtained high-quality datasets which highlighted Nr5a2’s binding to cis-regulatory elements, particularly in 2-cell and 8-cell embryos, and exhibited Nr5a2’s essential role in activating genes during the 4–8 cell stage transition. Their data showed that while Nr5a2 knockdown or knockout did not significantly impact ZGA, it led to impaired activation of 4–8 cell stage genes and resulted in embryonic arrest at the morula stage. Similarly, Festuccia et al. reported that Nr5a2 knockout embryos progressed beyond the two-cell stage but exhibited significant developmental delays and morphological abnormalities at the morula stage, highlighting its crucial role in morula development and first lineage specification [62,63]. We hypothesize that further studies using more specific inhibitors targeting Nr5a2 or the same genetic background mice may help resolve the debates about the role of Nr5a2.5. Pioneer Transcription Factors Utilize Retrotransposons Elements as Hubs for ZGA Regulation

Retrotransposons, which originated from ancient retroviruses, have colonized genomes and now make up nearly 40% of mammalian genomes [64]. Retrotransposons are categorized into LTR (long terminal repeat) retrotransposons, also referred to as ERVs (endogenous retroviruses), and non-LTR retrotransposons; non-LTR retrotransposons are also further divided into LINEs (long interspersed elements) and SINEs (short interspersed elements) [65,66,67,68]. Given that the transposition activities of retrotransposons may compromise genomic integrity and potentially lead to cancer and autoimmune diseases [68,69], retrotransposons are generally silenced in most somatic cells through various epigenetic modifications, including DNA methylation and repressive histone modifications, to reduce the risk of retrotransposition [70,71]. Counterintuitively, bursts of retrotransposon transcription usually occur during ZGA in preimplantation embryos [72,73]. From an evolutionary perspective, we believe that there is a delicate balance between the activation of retrotransposon transcription and genome integrity in the developing mammalian embryo. Facing the pressures of natural selection, retroviruses and hosts compromise with each other. Taking endogenous retroviruses as an example, on one hand, throughout evolutionary history, recombination between LTRs has led to the loss of internal ERV genes, resulting in solo LTRs and preventing the harmful expression of viral proteins within the host genome [74]; on the other hand, during the reprogramming window, early embryos tolerate a massive onslaught of ERV expression, using cis-regulatory elements located in LTRs to rewire the gene regulatory network [75]. It is well recognized that despite the colonization by selfish retroviruses, host genomes have successfully domesticated retrotransposon sequences for the benefit of the host species. This process is also known as co-option [76,77]. In other words, in the context of host early development, retrotransposons have evolved cis-regulatory sequences that recruit host-encoded factors, such as transcription factors, to ensure the expression of essential developmental genes during ZGA, ultimately achieving host co-option.

**Figure 1:**
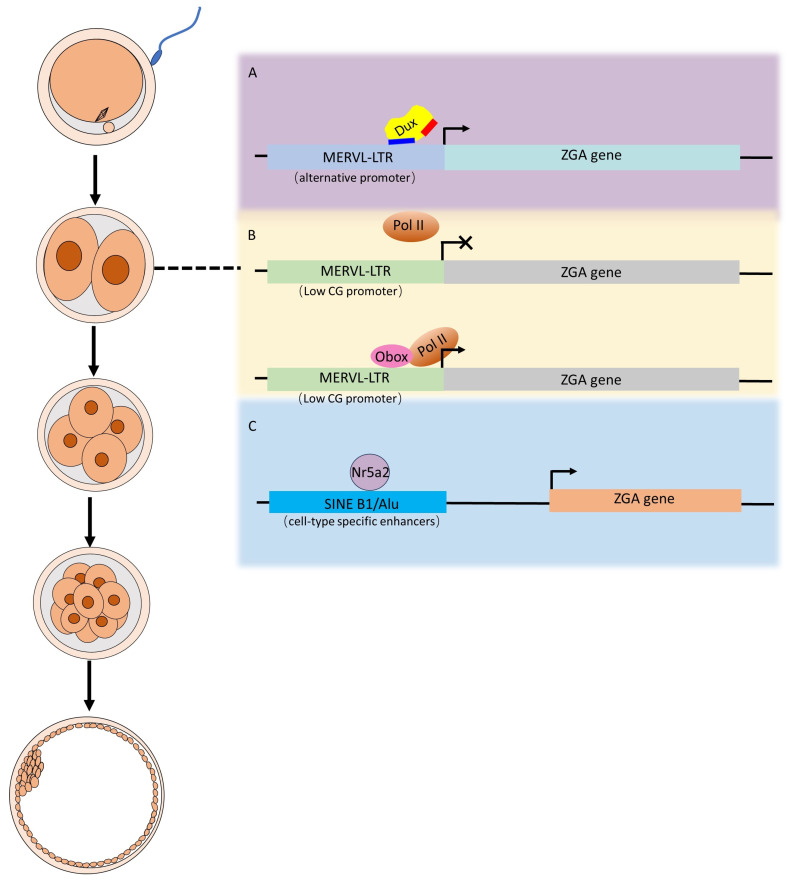
Scheme of pioneer transcription factors activating ZGA genes via retrotransposon elements. (**A**) ZGA gene activation by Dux. During ZGA, endogenous Dux is transiently expressed. Through the more divergent homeodomain (the first homeodomain) in DUX-C family proteins, Dux binds to the MERVL-LTR, which is co-opted by the host as an alternative promoter, thereby activating downstream ZGA genes. (**B**) ZGA gene activation by Obox. During ZGA, RNA pol II alone cannot locate CG-poor promoters of ZGA genes; therefore, Obox proteins are essential for recruiting RNA pol II to these promoters, which in turn activate downstream ZGA genes. (**C**) ZGA gene activation by Nr5a2. Nr5a2 specifically binds to its motif within a subtype of SINE B1/Alu transposable elements, which are prevalent in the enhancers of ZGA genes, ultimately activating downstream ZGA genes.

During ZGA, pioneer transcription factors, which reside at the top of the transcriptional hierarchy, initiate cascading effects on gene expression using retrotransposon elements as hubs. These three pioneer transcription factors (such as Dux, Obox and Nr5a2) discussed in this review all achieve their functions by targeting motifs embedded within retrotransposons, as shown in Figure 1. The proliferation of retrotransposon and its associated cis-regulatory elements facilitates the expansion of transcription factor binding sites (TFBSs) throughout the genome, thereby driving evolutionary innovations in regulatory mechanisms.

Dux has been pinpointed as a central inducer of ZGA, largely through binding to the MERVL LTR [17,18,19]. A significant portion of the transcriptome at the two-cell stage initiates transcription within the MERVL LTR. The MERVL LTR seems to have been co-opted by the host genome as an alternative promoter, driving the expression of genes related to the two-cell stage during ZGA [73]. Using CRISPRi (CRISPR interference), Yang et al. perturbed 2,485 (93%) MERVL-LTRs (MT2-Mm) insertions in two-cell embryos, and then observed downregulation of hundreds of ZGA genes [78]. Importantly, analysis with a conservative approach of unique reads indicated that Dux directly interacted with at least 53% of all MERVL-LTRs (MT2-Mm) and at least 37% of regions that showed increased ATAC sensitivity in 2C-like cells in mESCs. Furthermore, a consensus DUX-binding motif (WGATTYAATCW) was identified, and this motif showed significant enrichment in regions exhibiting increased ATAC sensitivity following Dux overexpression. Intriguingly, MERVL-LTRs (MT2_Mm) exhibited no significant DUX4-motif enrichment, and HERVL-LTRs (MLT2A1/2) displayed only minimal DUX-motif enrichment [18]. This phenomenon suggests that although both Dux in mice and DUX4 in humans are intronless and located in tandem arrays across multiple chromosomes, indicating functional conservation [23], they have evolved to be species-specific, possibly in response to ERVs.

Obox preferentially binds to and regulates ZGA genes via the Obox motif in mouse embryos. Ji et al. investigated the DNA binding sites of Obox proteins in early mouse embryos by overexpressing Obox genes (Obox1, Obox5, and Obox3) tagged with a Flag label. Using next-generation sequencing and bioinformatics tools, they confirmed that Obox was enriched at MERVL and B1/B2/B4 repetitive elements. In more detail, they identified two main de novo motifs that closely matched the known Obox binding motif (TAATCCC) [20,79]. In addition, these de novo motifs are often closely spaced at the binding sites, forming an extended binding motif (ACNCCTTTAATCCCAG), with Obox1 displaying the longest consecutive version of this motif (CCTTTAATCCCAG). Notably, approximately 95.9% of the extended Obox motif is located in the B1 element, resulting in stronger gene activation than the reported 7-bp motif in reporter assays in embryos. Additionally, Guo et al. discovered that Obox4 directly binds to MERVL and MERVK loci and activates specific MERVL and MERVK elements in a Dux-independent manner, positioning *Obox4* as a redundant gene relative to *Dux* in the context of ZGA [38]. It is noteworthy that in the context of ZGA, genes specific to the two-cell stage exhibit a pronounced enrichment for Obox transcription factor binding motifs at promoter regions, with an observed increase in motifs relative to genes activated at subsequent developmental stages, which indicates a distinct regulatory landscape compared to genes activated at subsequent developmental stages [20].

Nr5a2 has been identified as a crucial activator of ZGA in mouse embryos, primarily due to its binding to specific motifs within a subtype of SINE B1/Alu transposable elements located in the cis-regulatory regions of ZGA genes. Through exploring the enrichment of transcription factor motifs in the cis-regulatory regions of ZGA genes, a specific motif sequence was found to be prevalent in a significant majority of ZGA genes, containing variable pyrimidine/purine (YR) stretches. Particularly, the CA variant of the fifth YR stretch, YR5, is associated with enhanced chromatin accessibility and increased histone acetylation, suggesting its significant function during ZGA. In detail, the CA variant of the motif includes the consensus sequence TCAAGGCCA, which was identified as the Nr5a2 motif. By comparing the binding sites of Nr5a2 to the overlap with SINE B1 transposable elements, it was found that 70% of Nr5a2 peaks overlapped with SINE B1, suggesting that SINE B1 transposons are major targets for Nr5a2. The Nr5a2 motif identified in the peaks includes the sequence (A/G)(A/G)T upstream of consensus sequences, a pattern similarly observed in motif 1 within the SINE B1/Alu 5YR upstream of ZGA genes [21].

## 5. Conclusions

Pioneer transcription factors, such as Dux, Obox and Nr5a2, orchestrate a complex regulatory landscape during ZGA, which highlights their central role in developmental biology and their potential utility in improving reprogramming efficiency. Host genomes have successfully domesticated retrotransposon elements that served both as challenges and aids by evolving cis-regulatory sequences, which ensures the expression of crucial developmental genes during ZGA and achieves a symbiotic use of these retrotransposon elements. Specifically, pioneer transcription factors target these retrotransposon elements and employ these elements as regulatory hubs for ZGA, then these pioneer transcription factors reside at the top of a transcriptional hierarchy. Dissecting pioneer transcription factor functions not only provides insights into the fundamental processes of life but also opens avenues for the application of this knowledge in reproductive medicine, genetic research, animal breeding and regenerative medicine domains. In the future, the combination of Hi-C with super-resolution imaging techniques, which can provide insights into the dynamic organization of chromatin in the nucleus, may reveal further implications for the function of pioneer transcription factors in gene regulation [80].
